# Faecal Microbiota Divergence in Allopatric Populations of *Podarcis lilfordi* and *P. pityusensis*, Two Lizard Species Endemic to the Balearic Islands

**DOI:** 10.1007/s00248-022-02019-3

**Published:** 2022-04-28

**Authors:** Iris Alemany, Ana Pérez-Cembranos, Valentín Pérez-Mellado, José A. Castro, Antonia Picornell, Cori Ramon, José A. Jurado-Rivera

**Affiliations:** 1 Department of Biology, University of the Balearic Islands, Ctra., Valldemossa km 7’5, 07122 Palma, Balearic Islands Spain; 2 Department of Animal Biology, Universidad de Salamanca, Salamanca, Spain

**Keywords:** Faecal microbiota, *Podarcis lilfordi*, *Podarcis pityusensis*, Balearic Islands, Allopatric populations, Host-microbiome interactions

## Abstract

Gut microbial communities provide essential functions to their hosts and are known to influence both their ecology and evolution. However, our knowledge of these complex associations is still very limited in reptiles. Here we report the 16S rRNA gene faecal microbiota profiles of two lizard species endemic to the Balearic archipelago (*Podarcis lilfordi* and *P. pityusensis*), encompassing their allopatric range of distribution through a noninvasive sampling, as an alternative to previous studies that implied killing specimens of these IUCN endangered and near-threatened species, respectively. Both lizard species showed a faecal microbiome composition consistent with their omnivorous trophic ecology, with a high representation of cellulolytic bacteria taxa. We also identified species-specific core microbiota signatures and retrieved lizard species, islet ascription, and seasonality as the main factors in explaining bacterial community composition. The different Balearic *Podarcis* populations are characterised by harbouring a high proportion of unique bacterial taxa, thus reinforcing their view as unique and divergent evolutionary entities.

**Supplementary Information:**

The online version contains supplementary material available at 10.1007/s00248-022-02019-3.

## Introduction

Recent advances in sequencing technologies and analytical methodologies are improving our understanding of the microbiome in host evolution [1]. Early evolutionary conceptions considering animals and plants as autonomous entities are being challenged by the holobiont point of view, which also considers their numerous microbial symbionts and their genomes [2]. The sum of the genetic information of the host and its associated microbiota has been termed the hologenome [3, 4], whose variation can influence phenotypes upon which natural selection and/or genetic drift can operate [2]. Indeed, microbial communities can have a deep impact on host diversification by acting as environmental factors with selective effects [5], influencing many aspects of host evolutionary history such as adaptation to resource utilisation [6], resistance to pathogens [7], control of nutrient inputs [8], tissue and organ development [9], life history strategy [10] and behaviour [11], among many others. The relevance of this interaction is such that the evolutionary history of host species cannot be fully understood without addressing the study of its associated microbiota. However, despite the effort made over the last decade to characterise microbial-host associations, we still know relatively little about the evolutionary and ecological processes shaping them, particularly in non-model and non-captive organisms.

**Fig. 1:**
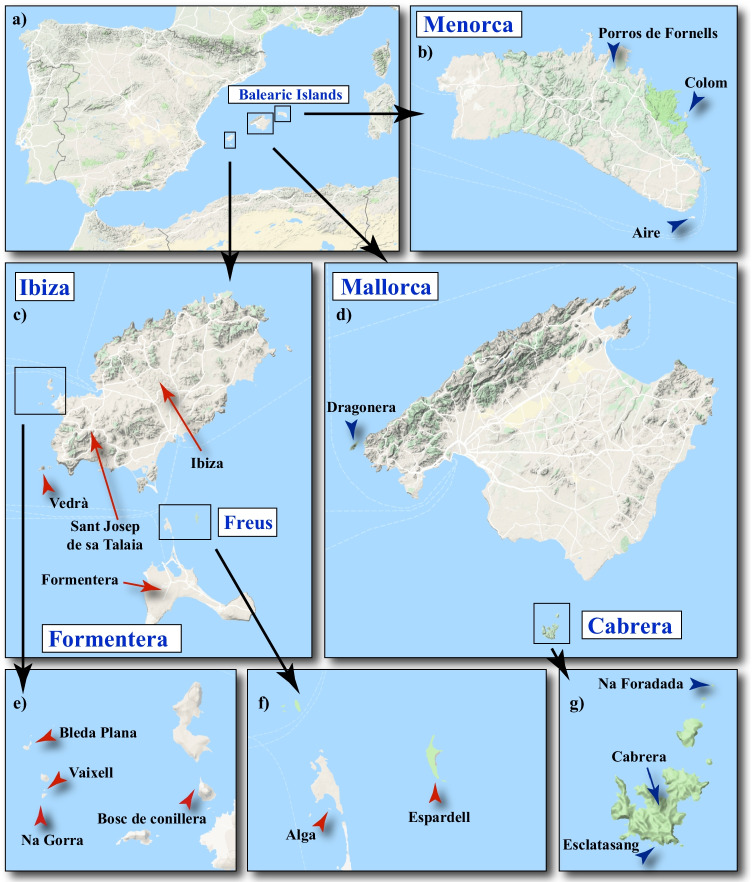
Maps of the Balearic archipelago showing the location of the sampled *Podarcis lilfordi* (blue arrows) and *P. pityusensis* populations (red arrows). Maps were obtained with Google Maps (Map data 2020 Google) using the function “get_map” in the package “ggmap” version 3.0.0.902 in R version 3.6.3

The lizard species endemic to the Balearic Islands, *Podarcis lilfordi* and *P. pityusensis*, represent an interesting model to study host-microbiota associations since they are sister taxa with a nonoverlapping distribution, consisting of multiple allopatric populations restricted to coastal islands and islets of Mallorca, Menorca, and Cabrera in the case of *P. lilfordi*, and the main islands of Ibiza and Formentera in *P. pityusensis*. *Podarcis lilfordi* became extinct on both the main Mallorca and Menorca islands due to the anthropic introduction of foreign predators and/or competitors [12, 13]. The feeding ecology of both species is well-known [14–17] and is marked by the scarcity and unpredictability of diet resources in their isolated habitats, which probably determined the adoption of omnivory [17]. Balearic lizards are active foragers exploiting a wide range of animal prey, plant tissues, carrion, and marine subsidies, showing even low levels of cannibalism of juvenile conspecifics [17]. On the other hand, the phylogeographic relationships of the group have also been profusely studied, revealing an evolutionary origin linked to eustatic sea-level changes associated with the reflooding of the Mediterranean at the end of the Messinian Salinity Crisis that occurred 5.33 Ma ago [18–20]. The Menorca lineage represents the earliest cladogenetic event (2.6 Ma) within *P. lilfordi*, followed by the differentiation of the West Mallorca lineage (2.0 Ma), Cabrera (1.2 Ma), and the remaining populations in northern and southern Mallorca islets [20] (see Fig. 1 for island configuration in the Balearic archipelago). Regarding *P. pityusensis*, the Ibiza and Formentera populations have been reported to be genetically distinct, with a divergence estimated to have occurred *ca.* 0.111–0.295 Ma ago [21].

Although lizards are a globally distributed and species-rich group within vertebrates [22, 23], they have been largely overlooked in terms of gut microbiota [24]. More than 90% of such studies within vertebrate hosts have been carried out in mammal species, with reptiles being the least investigated group [25]. To date, a single study on the microbiota of the Balearic lizards has been addressed [26]. This work was focused on a very limited portion of the distribution of one of the species (seven Menorcan populations of *P. lilfordi*) and based on sex-biased sampling (30 males and 3 females) from a single season (summer) using methodologies that imply killing specimens of this endangered (IUCN) species. The study reported a significant effect of the geographical distribution (islet) in explaining the 13.5% of the bacterial community variation and a very low bacterial uniqueness in each lizard population, suggesting the retention of the ancestral mainland microbial pool and the occurrence of stochastic population processes as the main factors shaping the gut microbiota.

Here we aim to further explore the evolutionary history of these two endemic lizard species by using Illumina 16S rRNA sequencing to characterise their faecal microbiota across different seasons of the year and through a sex-balanced and noninvasive sampling. Specifically, we address the following questions: (i) what is the gut bacterial composition of these two endemic species? (ii) Are there species-specific microbiome core signatures? (iii) Do microbial communities mirror the allopatric distribution of their respective lizard populations? (iv) What is the degree of uniqueness of each *Podarcis* population from a microbiome perspective? (v) Do the microbiome communities reflect the trophic ecology of the host populations?

## Materials and Methods

### Sampling

The sampling design included 17 localities encompassing the distribution range of *P. lilfordi* and *P. pityusensis* in the Balearic archipelago (Fig. 1, Table 1, and Table S1a). A total of 242 faecal samples from *P. lilfordi* (140) and *P. pityusensis* (102) were collected between the spring of 2016 and the autumn of 2017. Specimens were captured by noosing and fresh faeces were collected in absolute ethanol vials directly from the animals before releasing them back to their habitats. Individuals were sexed based on the number and size of the femoral pores [27]. The samples were immediately preserved at 4 °C in the field and upon arrival at the laboratory, where they were stored at − 20 °C until DNA extraction.

**Table 1:** Sampling localities and associated metadata

				Female	Male	Season	Female	Male	Season
***P. lilfordi***	CABRERA	Cabrera	Cabrera	6	7	Autumn	12	16	Summer
		Esclatasang	Esclatasang	4	4	Autumn	6	4	Summer
		Na Foradada	Na Foradada	2	5	Autumn	2	8	Summer
	MALLORCA	Dragonera	Dragonera	9	8	Summer	2	8	Autumn
	MENORCA	Aire	Aire	-	1	Summer	5	5	Spring
		Colom	Colom	3	3	Summer	6	4	Spring
		Porros de Fornells	Porros	-	-	-	4	6	Spring
***P. pityusensis***	FREUS	Alga	Alga	2	3	Summer	5	5	Summer
		Espardell	Espardell	5	-	Summer	7	6	Spring
	FORMENTERA	Formentera	Formentera	-	-	-	4	6	Spring
	IBIZA	Bleda Plana	Bleda	-	1	Summer	-	-	-
		Bosc de conillera	Bosc	4	3	Spring	-	-	-
		Na Gorra	Na Gorra	13	11	Summer	5	5	Spring
		Sant Josep de sa Talaia	St. Josep	1	2	Spring	-	-	-
		Ses Salines	Ibiza	2	2	Spring	-	-	-
		Vaixell	Vaixell	-	-	-	5	3	Summer
		Vedrà	Vedrà	-	2	Summer	-	-	-

### DNA Extraction, 16S rRNA Library Preparation, and Sequencing

Total DNA was extracted from individual samples using the ISOLATE Fecal DNA kit (Bioline, London, UK) following the manufacturer protocol, and their concentrations were quantified using Qubit fluorometric quantitation (ThermoFisher, Foster City, CA, USA). For cost-effective reasons and given that our study seeks to describe the bacterial composition of each lizard population as a whole, samples from each island/islet with matching sex, season, and collecting year were pooled in equimolar concentrations, obtaining a final volume of 30 μl at 20 ng/μl per sample (see Table 1 for details on the number of faecal samples pooled per location). A total of 48 samples were submitted to the Roy J. Carver Biotechnology Center (University of Illinois, USA) for amplification of the V4 region of 16S rRNA in a microfluidic high-throughput multiplexed PCR platform (Fluidigm). The primer set 515F (5′-GTGCCAGCMGCCGCGGTAA-3′) and 806R (5′-GGACTACHVGGGTWTCTAAT-3′) [28] were used, and CS1 and CS2 Fluidigm universal tags, barcode labels specific to each sample and Illumina adapters i5 and i7. The resulting amplicons were validated on a fragment analyzer (Agilent) using the HS NGS kit (DNF-474–33). Sequencing was conducted on an Illumina MiSeq v2 platform yielding 2 × 250 bp paired-end reads.

### Sequence Analyses

QIIME2 version 2020.2 [29] was used for read demultiplexing and subsequent filtering and denoising using the DADA2 pipeline [30]. Sequences were grouped into amplicon sequence variants (ASVs or 100% identity OTUs), and the taxonomic assignment was performed using the *q2-feature-classifier* plugin with a pre-trained naive Bayes classifier [31] implemented in QIIME2 against the SILVA database release 132 [32]. ASVs identified as chloroplasts or mitochondria, and those with undetermined phylum annotation were excluded for downstream analyses. Sequences were aligned with MAFFT [33] under the default FFT-NS-1 algorithm, and a phylogenetic tree was inferred with FastTree [34] using the QIIME2 plugin *align-to-tree-mafft-fasttree*.

### Datasets

To avoid comparing samples from different seasons and to dissect the potential effect of the host lizard species (*P. lilfordi* / *P. pityusensis*) and geography (islet) on the faecal microbiota, we subdivided the data into three datasets by collecting seasons: spring, summer, and autumn. In addition, we further explored the data by analysing those samples with matching collecting seasons: spring + summer dataset [Espardell (Fig. 1f), Na Gorra (Fig. 1e), Aire and Colom (Fig. 1b)], and summer + autumn dataset [Dragonera (Fig. 1d), Cabrera, Esclatasang and Na Foradada (Fig. 1g)].

### Diversity Analyses

Rarefaction curves were explored using the *rarecurve* function implemented in the R package *vegan* [35]. The ASV table was rarefied using the *rarefy_even_depth* option in the R package *phyloseq* [36] by subsampling the data to the even depth defined by the minimum number of sequences per sample. This value was set to 10,815 in the *P. lilfordi* dataset and 19,159 in the case of *P. pityusensis*. When analysing the dataset of both species combined, the lowest value was chosen.

Alpha diversity was measured through the estimation of the absolute number of observed ASVs, Shannon and Simpson indexes in *phyloseq*, and Faith’s phylogenetic diversity (PD) in the *picante* R package [37]. Differences in terms of alpha diversity at both inter- and intra-specific levels were assessed using the Kruskal–Wallis test.

Although the pooled nature of the samples erases the interindividual variability within each island/islet, we carried out exploratory beta diversity analyses as a proxy to understand the extent of change in bacterial community composition across lizard populations. To delve into it, we performed a permutational multivariate analysis of variance (PERMANOVA) using the *adonis* function implemented in the *vegan* package and based on both unweighted and weighted UniFrac and Bray–Curtis distance matrices [35]. Specifically, we tentatively explored potential correlations between community microbiota distances and the categorical variables associated to our samples, namely species, sex and islet. The later variable was also explored at a broader scale by assigning each sampling locality (i.e., islet/population) to a main island district in the Balearic archipelago (Mallorca, Menorca, Cabrera, Ibiza, or Formentera). The unweighted UniFrac matrices were also used to perform PCoA ordination analyses. Finally, we tested for correlation between the variable number of faecal pellets pooled per sample and microbiome community composition distances (Unweighted and Weighted UniFrac matrices) by conducting Mantel tests based on the Spearman correlation method and performing 9999 replicates.

### Core Microbiota

Shared microbiome taxa were independently investigated for each one of the two lizard species with the *core members* function of the *microbiome* R package [38], setting the prevalence threshold at 90%. The intersection of the resulting sets was interpreted as the core of the lineage *P. lilfordi* + *P. pityusensis*, and their differences were, respectively, considered the core signature of each one of the two *Podarcis* species.

### Uniqueness and Shared Microbial Taxa

UpSetR package [39] was used to calculate both unique and shared ASVs by population and/or species.

## Results

### Sequence Summary

We obtained 16S rRNA sequence data from 48 faecal pools representing 140 specimens of *P. lilfordi* and 102 of *P. pityusensis* (Fig. 1 and Table 1). After the quality filtering stage and the removal of features identified as chloroplasts, mitochondria, or Eukaryota and those with undetermined phylum annotation, the dataset was reduced to 3,488,522 high-quality reads with an average value of 72,678 reads per sample (range = 11,107–336,352). The reads could be ascribed to 3542 ASVs representing 20 phyla, 41 classes, 93 orders, 190 families, and 425 genera of microbiome taxa. A total of 369 ASVs were unique to *P. lilfordi* and 766 to *P. pityusensis*. The sampling depth values used in the rarefaction analyses allowed us to get most of the diversity of the samples, as shown by the asymptotic trend of the resulting curves (Fig. S1). The taxonomy of rarefied and the non-rarefied ASV tables are provided as Table S1b, c, and d.

### Bacterial Taxonomic Composition

**Fig. 2:**
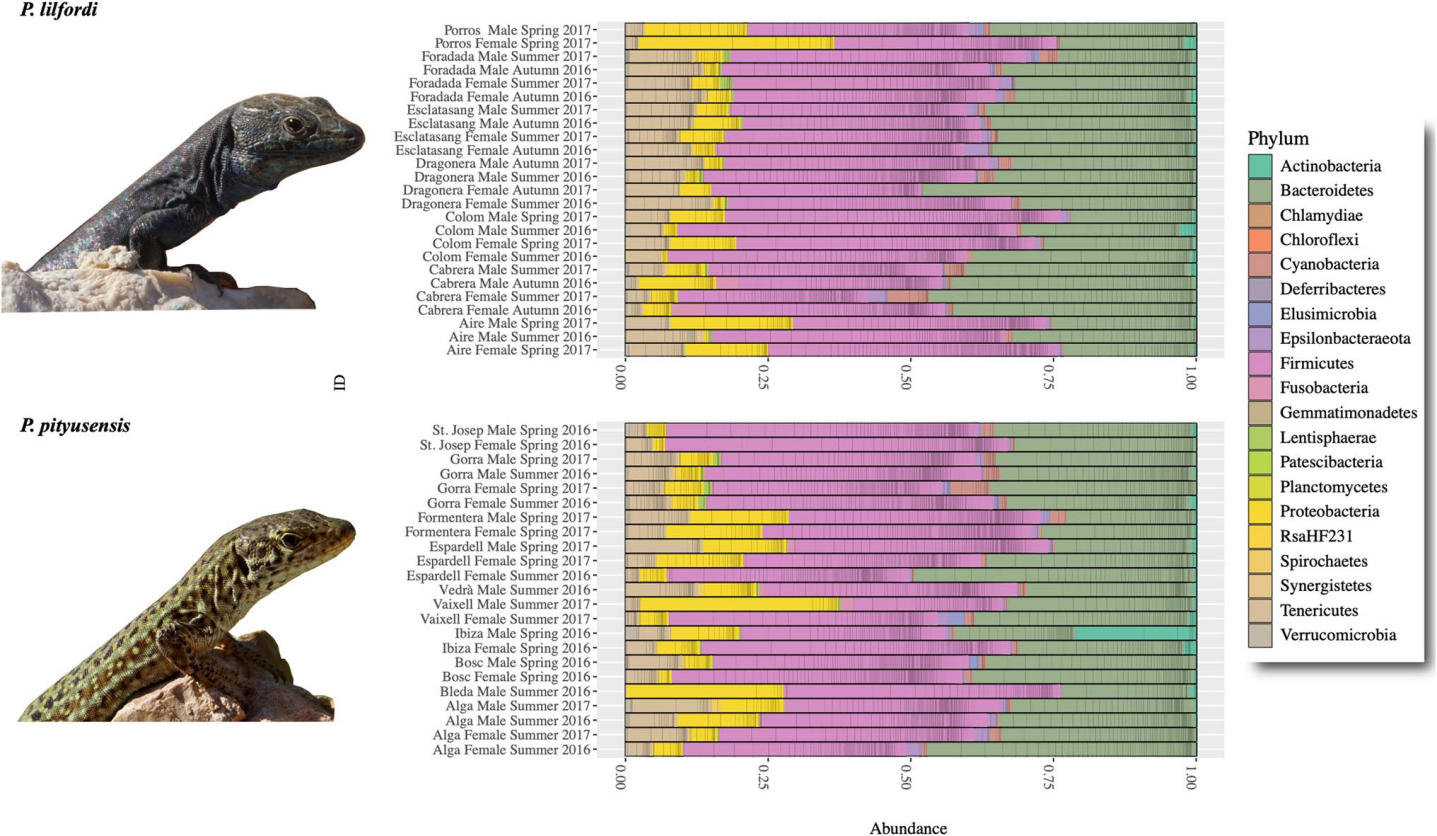
Microbiota composition at the phylum level per sample

The taxonomic distribution of the bacterial assemblages was variable among samples (Figs. 2, S2, S3, S4 and Table S2). Four phyla (Fig. 2 and Fig. S2) represented altogether 95% of the microbiota (Table S2): Bacteriodetes (average = 33%; range = 21.3–49.1%), Firmicutes (average = 45.1%; range = 33.4–59.9%), Proteobacteria (average = 8.9%; range = 0.6–34.5%), and Tenericutes (average = 7.6%; range = 0.1–14.9%).

The faecal microbiota of both lizard species was dominated by two orders (Fig. S3): Clostridiales (average = 38.2%; range = 14.2–56.2%; Firmicutes) and Bacteroidales (average = 33.1%; range = 21.5–48.8%; Bacteroidetes). Families Bacteroidaceae (average = 16.9%; range = 4.6–27.4%) and Rikenellaceae (average = 6.4%; range = 0.5–16.6%) showed the highest relative abundance within Bacteroidetes, while Lachnospiraceae.

(average = 13%; range = 2.1–33.9%), Clostridiales vadinBB60 group (average = 10.4%; range = 0.1–23.9%), and Ruminococcaceae (average = 8.5%; range = 2–16.9%) were the most abundant within Firmicutes.

### Alpha Diversity

Faecal samples from *P. lilfordi* and *P. pityusensis* showed similar average alpha diversity values (Table 2) with no significant differences between both lizard species (Table S3). At the population level, most indicators reported Es Vedrà as the location with the highest average microbiome diversity values, followed by Na Gorra, Bosc, Espardell, and Formentera, all of them belonging to *P. pityusensis*. Oppositely, two *P. pityusensis* populations received the lowest diversity values (Bleda and Vaixell). *Podarcis lilfordi* populations were scored with relatively moderate values for all indexes, standing out Na Foradada, Cabrera, and Esclatasang as the most diverse locations, while the Menorcan populations of Colom and Porros islets received the lowest alpha diversity values (Table 2).

**Table 2:** Mean (minimum–maximum) *P. lilfordi* and *P. pityusensis* alpha diversity indexes by population

Species	Localization	Dataset (by Island/Islet)	Shannon	Chao1	Observed	Simpson	PD
*P. lilfordi*	CABRERA ARCHIPELAGO	Cabrera	4.87 (4.65–5.32)	426.18 (314.14–704.03)	408.75 (311–649)	0.98 (0.98–0.99)	19.87 (16.53–27.34)
		Esclatasang	4.88 (4.80–5.00)	338.55 (306.00–388.52)	336 (306–383)	0.99 (0.98–0.99)	19.28 (18.31–21.01)
		Na Foradada	4.93 (4.31–5.19)	487.91 (270.94–634.00)	462.50 (270–592)	0.98 (0.97–0.99)	22.82 (17.18–26.42)
	MALLORCA	Dragonera	4.69 (3.72–5.09)	378.77 (156.00–536.46)	362.75 (156–495)	0.97 (0.93–0.99)	18.23 (10.48–22.94)
	MENORCA	Aire	4.72(4.50–4.96)	280.62 (201.00–331.00)	279.67 (201–331)	0.98 (0.98–0.99)	15.72 (12.63–17.94)
		Colom	4.41 (4.12–4.61)	339.89 (253.05–435.49)	308 (241–362)	0.97 (0.97–0.98)	15.40 (12.95–17.79)
		Porros	4.48 (4.14–4.82)	316.50 (310.78–322.23)	310.50 (303–318)	0.97 (0.96–0.98)	15.16 (14.96–15.36)
*P. pityusensis*	FREUS	Alga	4.84 (4.72–5.01)	483.15 (313.24–733.46)	440.50 (310–637)	0.98 (0.98–0.98)	20.89 (17.15–26.57)
		Espardell	4.96 (4.78–5.05)	512.11 (442.57–610.94)	466.33 (432–517)	0.98 (0.98–0.99)	20.75 (19.64–21.88)
	FORMENTERA	Formentera	4.97 (4.90–5.03)	397.42 (375.34–419.50)	393.50 (375–412)	0.99 (0.99–0.99)	19.46 (18.90–20.01)
	IBIZA	Bleda	3.56	194.08	186	0.92	10.44
		Bosc	5.01 (4.99–5.04)	520.61 (507.70–533.53)	486 (478–494)	0.99 (0.99–0.99)	20.85 (19.72–21.97)
		Ibiza	4.53 (4.47–4.59)	515.18 (425.65–604.71)	452.50 (411–494)	0.97 (0.97–0.97)	21.6 (21.39–21.81)
		Na Gorra	5.24 (5.05–5.41)	539.22 (452.37–652.80)	518.75 (444–611)	0.99 (0.98–0.99)	24.37 (22.56–26.51)
		St. Josep	4.48 (4.22–4.73)	265.7 (264.25–267.14)	264 (261–267)	0.98 (0.97–0.98)	14.41 (14.06–14.75)
		Vaixell	4.17 (3.64–4.70)	285.03 (241.06–329.00)	279.50 (238–321)	0.93 (0.89–0.98)	16.19 (13.75–18.63)
		Vedrà	5.19	799.57	687	0.99	27.31
Dataset (by Season)							
*P. lilfordi*		Spring	4.62 (4.14–4.96)	324.15 (309.32–361.7)	314.67 (296–333)	0.98 (0.96–0.99)	15.95 (14.97–17.94)
		Summer	4.78 (4.13–5.32)	402.81 (201.00–704.03)	381.36 (201–649)	0.98 (0.97–0.99)	19.43 (12.63–27.34)
		Autumn	4.74 (3.72–5.19)	373.04 (156.00–634.00)	361.13 (156–592)	0.98 (0.93–0.99)	18.80 (10.48–26.42)
*P. pityusensis*		Spring	4.83 (4.22–5.11)	446.76 (264.25–610.94)	419.33 (261–517)	0.98 (0.97–0.99)	20.03 (14.06–22.57)
		Summer	4.75 (3.64–5.41)	475.11 (194.08–799.57)	438.91 (186–687)	0.97 (0.89–0.99)	20.50 (10.40–27.30)
Dataset (by Species)							
*P. lilfordi*		all *P. lilfordi* samples	4.73 (3.72–5.32)	374.40 (156.00–704.03)	358.88 (156–649)	0.98 (0.93–0.99)	18.40 (10.48–27.34)
*P. pityusensis*		all *P. pityusensis* samples	4.79 (3.56–5.41)	460.32 (194.08–799.57)	428.70 (186–687)	0.98 (0.89–0.99)	20.26 (13.75–26.51

**Fig. 3:**
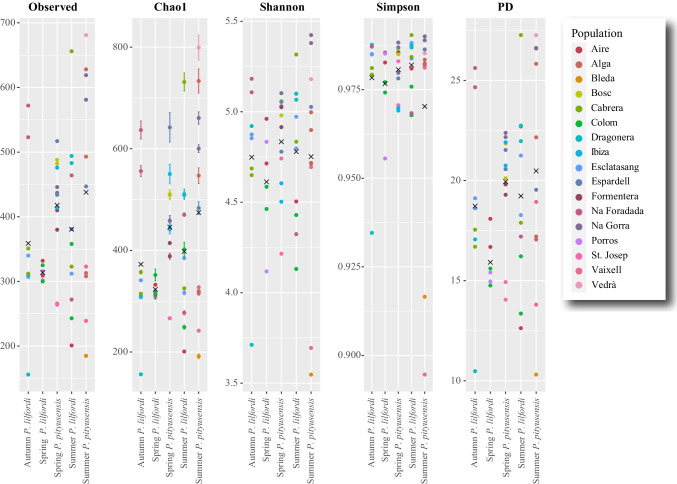
Alpha diversity indexes (observed number of ASVs, Chao1, Shannon, Simpson, and Faith’s phylogenetic diversity) from the datasets consisting of samples merged by collecting season and lizard species. Average index values are indicated by “X” symbols

When considering only samples collected in the same season and lizard species, all datasets showed similar average diversity indexes (Table 2) and no significant differences among samples within the same dataset (Table S4). However, inter-dataset comparisons revealed significant differences in terms of alpha diversity indices (observed number of ASVs, Chao1, and phylogenetic diversity) between the spring samples of *P. lilfordi* and those of *P. pityusensis* (Table S3 and Fig. 3), being *P. lilfordi* the species exhibiting the lowest diversity values (Table 2; Fig. 3).

### Beta Diversity

Mantel tests for correlation between differences in terms of the number of faecal pellets pooled on each sample and microbiome community distances yielded non-significant results regardless of whether the analyses were based on the Unweighted or the Weighted UniFrac matrices from the entire dataset (i.e., all 48 samples) or from each of the subsets by season (spring, summer, and autumn datasets) (Table S5a–i, Table S6). We therefore tentatively explored if the microbiota community composition from the analysed pooled samples varied across species, island/islet, main island district, season and/or sex. PERMANOVA analyses yielded significant microbiome composition differences between the two lizard species and, also, in relation to the geographical distribution of the samples, regardless the collecting season and of whether the analyses were based on weighted or unweighted UniFraq matrices (Table 3). Among the significant variables, island/islet was reported as the most important source of variance in explaining bacterial community composition, with *R*^2^ values ranging from 0.36 to 0.48, followed by the main island district variable (*R*^2^ range: 0.06 to 0.21). When analysing the datasets conformed by samples with matching collecting seasons (i.e., spring + summer and summer + autumn datasets), PERMANOVA tests consistently retrieved the species, the island/islet, and the main island district as significant variables in explaining the microbial community composition of the samples, and in addition both the season variable and the interaction of population:season were also retrieved as significant in some of the analyses (Table 3).

**Table 3:** Results of PERMANOVA analyses based on both unweighted and weighted UniFrac distance matrices for each season. Significant *p*-values (< 0.05) are highlighted in bold

Dataset	Variable	Unweighted UniFrac						Weighted UniFrac					
		Df	Sums of sqs	Mean sqs	F. model	R2	Pr (> F)	Df	Sums of sqs	Mean sqs	F. model	R2	Pr (> F)
Spring	Species	1	0.43	0.43	2.63	0.11	**0.001**	1	0.00	0.00	1.85	0.08	**0.019**
	Sex	1	0.17	0.17	1.02	0.04	0.301	1	0.00	0.00	1.15	0.05	0.261
	Main island district	1	0.25	0.25	1.51	0.06	**0.016**	1	0.00	0.00	1.48	0.06	0.096
	Population	6	1.75	0.29	1.79	0.45	**0.001**	6	0.02	0.00	1.92	0.48	**0.001**
	Residuals	8	1.30	0.16	NA	0.33	NA	8	0.01	0.00	NA	0.33	NA
	Total	17	3.90	NA	NA	1	NA	17	0.04	NA	NA	1	NA
Summer*	Species	1	0.50	0.50	3.10	0.09	**0.001**	1	0.01	0.01	2.73	0.08	**0.011**
	Sex	1	0.23	0.23	1.45	0.04	**0.029**	1	0.00	0.00	1.85	0.06	0.058
	Main island district	3	1.14	0.38	2.37	0.21	**0.001**	3	0.02	0.01	2.40	0.21	**0.007**
	Population	7	2.17	0.31	1.93	0.40	**0.001**	7	0.03	0.00	1.84	0.38	**0.030**
	Residuals	9	1.45	0.16	NA	0.26	NA	9	0.02	0.00	NA	0.27	NA
	Total	21	5.50	NA	NA	1	NA	21	0.07	NA	NA	1.00	NA
Autumn**	Sex	1	0.15	0.15	0.86	0.10	0.756	1	0.00	0.00	1.34	0.13	0.211
	Main island district	1	0.28	0.28	1.59	0.19	**0.030**	1	0.00	0.00	2.06	0.20	**0.023**
	Population	2	0.52	0.26	1.51	0.36	**0.020**	2	0.01	0.00	2.03	0.39	**0.014**
	Residuals	3	0.52	0.17	NA	0.35	NA	3	0.00	0.00	NA	0.29	NA
	Total	7	1.47	NA	NA	1	NA	7	0.02	NA	NA	1	NA
Spring + Summer	Species	1	0.59	0.59	4.14	0.19	**0.001**	1	0.01	0.01	5.04	0.17	**0.001**
	Main island district	1	0.43	0.43	3.02	0.13	**0.001**	1	0.01	0.01	5.62	0.19	**0.001**
	Population	1	0.41	0.41	2.84	0.13	**0.001**	1	0.01	0.01	3.69	0.12	**0.004**
	Season	1	0.22	0.22	1.55	0.07	0.068	1	0.00	0.00	3.92	0.13	**0.006**
	Population: season	3	0.69	0.23	1.60	0.22	**0.017**	3	0.01	0.00	1.96	0.19	**0.021**
	Residuals	6	0.86	0.14	NA	0.27	NA	6	0.01	0.00	NA	0.20	NA
	Total	13	3.20	NA	NA	1	NA	13	0.04	NA	NA	1	NA
Summer + Autumn	Main island district	1	0.32	0.32	2.17	0.11	**0.001**	1	0.01	0.01	2.38	0.12	**0.030**
	Population	2	0.69	0.34	2.30	0.22	**0.001**	2	0.01	0.01	2.22	0.22	**0.006**
	Season	1	0.24	0.24	1.58	0.08	**0.016**	1	0.00	0.00	1.54	0.08	0.111
	Population: season	3	0.63	0.21	1.40	0.20	**0.008**	3	0.01	0.00	1.31	0.19	0.157
	Residuals	8	1.20	0.15	NA	0.39	NA	8	0.02	0.00	NA	0.39	NA
	Total	15	3.07	NA	NA	1	NA	15	0.05	NA	NA	1	NA

^*^Populations represented by a single-sequenced sample (i.e., Bleda Plana and Vedrà in the summer dataset) were dropped out from PERMANOVA analyses

^**^The autumn dataset contained only *P. lilfordi* samples and therefore was not tested for the “species” variable

**Fig. 4:**
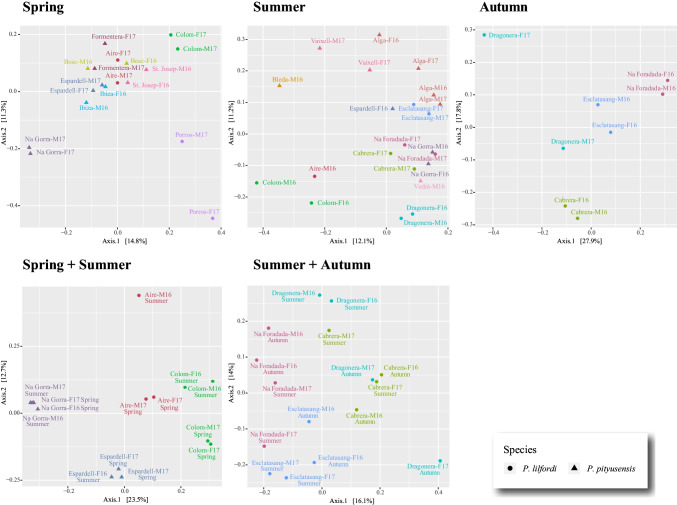
Principal coordinate analysis based on the unweighted UniFrac microbiome distances from the five analysed datasets (see main text for details) highlighting the effect of sample source (dot colours) and lizard species (dot shapes). The sex of the samples (M = males; F = females) and their respective collecting years (16 = 2016; 17 = 2017) are indicated in the text labels accompanying de dots (e.g., M-16 stands for males sampled in 2016)

The ordination analysis of the bacterial community distances by island/islet and species through principal coordinate analysis (PCoA) based on the unweighted UniFrac matrices from the samples collected in the same season of the year resulted in the clustering of the samples from the same location with few exceptions (Fig. 4).

### Core Composition

The number of core ASVs present in at least 90% of the samples of *P*. *lilfordi* and *P. pityusensis* was, respectively, 24 and 27, 13 of which were shared between both species (Table S7a, b). Exclusive *P*. *lilfordi* core ASVs included species from genera *Alistipes*, *Anaeroplasma*, *Bacteroides*, *Desulfovibrio*, *Helicobacter*, and *Parabacteroides*, while *P. pityusensis* unique core taxa were represented by ASVs from genera *Bacteroides*, *Coprococcus 3*, *Dielma*, *Erysipelatoclostridium*, *Eubacterium*, *Odoribacter*, *Parabacteroides*, *Robinsoniella*, and *Romboutsia*. The shared core taxa between both species comprised 3 phyla, 4 classes, 4 orders, 7 families, and 7 genera. *Bacteroides* were the most abundant genera, followed by *Odoribacter*. The common core species was *Desulfovibrio desulfuricans*.

### ASV Uniqueness

We explored the presence/absence of population-specific ASVs/taxa (Table 4). A total of 2042 ASVs were found in *P. lilfordi*, while 2384 were detected in *P. pityusensis*. Within *P. lilfordi*, ASV uniqueness at the population level ranged from 13.8 to 32.77% (Aire and Porros, respectively). Consistently, *P. pityusensis* also showed a high proportion of population-specific ASVs (range 7.02 to 25.17%; St. Josep and Vaixell, respectively). In total, we detected 1050 population-specific ASVs in *P. lilfordi* and 1126 in *P. pityusensis*.

**Table 4:** Proportion of uniqueness and shared OTUs by the studied *Podarcis* populations

P. lilfordi	CABRERA	Cabrera	836	163 (19.50)	80.50
		Esclatasang	703	139 (19.77)	80.23
		Na Foradada	944	236 (25.00)	75.00
	MALLORCA	Dragonera	750	129 (17.20)	82.80
	MENORCA	Aire	558	77 (13.80)	86.20
		Colom	625	150 (24.00)	76.00
		Porros	476	156 (32.77)	67.23
		Total	2042	1050	
P. pityusensis	FREUS	Alga	940	210 (22.34)	77.70
		Espardell	869	160 (18.41)	81.59
	FORMENTERA	Formentera	579	66 (11.40)	88.60
	IBIZA	Bleda	186	16 (8.60)	91.40
		Bosc	693	67 (9.67)	90.33
		Ibiza	719	151 (21.00)	79.00
		Na Gorra	988	198 (20.04)	79.96
		St. Josep	399	28 (7.02)	92.98
		Vaixell	453	114 (25.17)	74.83
		Vedrà	687	116 (16.89)	83.11
		Total	2384	1126	

## Discussion

### Non-Destructive Sampling as an Alternative to Killing Threatened Podarcis Lizards for Microbiome Analyses

Here we report the first characterisation of the *Podarcis pityusensis* faecal microbiota and expand the geographic sampling of *P. lilfordi* (Baldo et al. [26] only analysed Menorcan populations) through a noninvasive approach that avoids killing specimens. Although our results and those reported by Baldo et al. [26] from guts of killed *Podarcis* individuals could be affected by the implementation of different Illumina platforms (v2 vs v3), the length of the sequenced fragments (2 × 250 vs 2 × 300), and target 16S rRNA region (V4 vs V3-V4), the comparison between matching localities from both studies (Aire, Colom, and Porros) yields similar results at major taxonomic levels (this study/[26]/shared: phyla 16/12/12, classes 26/25/14, orders 59/37/25, families 101/50/30, genera 179/68/37). Although the different methodologies implemented in both studies make it difficult to carry out differential abundance analyses, comparing our results to past studies of the same lizard species shows that faecal samples can recover a high proportion of the taxa seen in gut samples. Faecal sampling has been used in microbiome studies on reptiles (e.g., [40–42]) and has been demonstrated to provide a comprehensive understanding of their hindgut bacterial communities [24]. Consistently, our findings reinforce this view and suggest that there is no need to kill lizards to characterise their microbiota at least at high taxonomic levels, which is even more relevant when dealing with species included in the IUCN Red List, such as the Balearic lizards *P. lilfordi* (endangered) and *P. pityusensis* (near threatened).

### The Faecal Microbiota of the Balearic Podarcis

Most lizard species are regarded as primarily feeding on invertebrates (e.g., [43, 44]), and less than 2% of the species are known to exploit plants as their sole food source [45]. However, many species do consume plant tissues under conditions of prey scarcity, a behaviour that is more frequently observed in island taxa (see [46] and references therein), including the *Podarcis* species endemic to the Balearic archipelago [16, 47]. Even though herbivory is rare among reptiles, they are known to perform hindgut fermentation either in the cecum or in the large colon/intestine, as also occurs in many lineages of mammals and birds [48, 49]. Indeed, there exists evidence of rapid adaption to plant diet through the acquisition of cecal valves, which slow down food flow and act as fermenting chambers in other *Podarcis* lizard species from the Mediterranean [50]. Like other vertebrates, reptiles lack the endogenous glycoside hydrolases needed to effectively hydrolyse and to ferment the complex plant polymers found in celluloses and hemicelluloses [51], and therefore rely on specialised bacterial communities [52]. Previous studies on the gastrointestinal microbiota of herbivore reptiles have reported a high prevalence of cellulolytic bacteria belonging to the phyla Bacteroidetes and Firmicutes (e.g., [24, 52, 53]), a pattern that is also matched by our results. Most Firmicutes taxa detected in the faecal microbiota of both *P. lilfordi* and *P. pityusensis* belong to class Clostridia order Clostridiales, a lineage of bacteria that includes most Firmicutes in both mammalian and reptilian herbivores [53]. Within Clostridiales, our results are highly represented by families Lachnospiraceae and Ruminococcaceae, both typically found in the gut microbiota of animals and known to decompose complex plant material [54]. Examples of Ruminococcaceae genera reported here with known implications in fibre digestion include *Oscillospira* and *Ruminoccocus* [55], represented in our dataset by 4 and 26 distinct ASVs, respectively (Table S1). Regarding Bacteroidetes, both *Podarcis* species showed a high proportion of microbes from families Bacteroidaceae, Porphyromonadaceae, Rikenellaceae, and Odoribacteraceae, all of them previously reported from the gut microbiota of reptiles with herbivorous habits (e.g., [26, 40, 56]). Within Bacteroidaceae, our results have reported the presence of 104 different ASVs from genus *Bacteroides* (Table S1), a lineage of active degraders of plant material [57, 58]. The gut microbiotas of *P. lilfordi* and *P. pityusensis* also include bacterial lineages that are consistent with their omnivorous ecology. This would be the case of the members of phylum Deferribacteres, which seem to be absent in the microbiota of generalist herbivorous lizards but is found in species that exploits both animal preys and plants [24], or the Clostridiales vadinBB60 group, a Firmicutes lineage with a high prevalence in carnivorous reptiles [59]. The relatively low prevalence of Actinobacteria and Proteobacteria in *Podarcis* samples is consistent with previous reports from other lizard species [24]. Within the latter our results report the presence of 20 distinct ASVs from *Desulfovibrio* (Table S1), a genus whose abundance has been correlated with fibre digestion [60] and that may play an important role in herbivorous lizards [24].

### Species-Specific Microbiome Signatures

Our exploratory beta diversity analyses pointed to significant differences in bacterial community composition between *P. lilfordi* and *P. pityusensis* samples regardless of the dataset (spring, summer, or spring + summer) and the input matrix (weighted or unweighted UniFrac) (Table 3). Such differences are also evident at the level of alpha diversity, where we have found significant differences between samples of both species collected in spring, being *P. pityusensis* the species exhibiting the highest diversity values. Although these results should be interpreted with caution due to the pooled nature of the samples, there is evidence that microbiota analyses based on mixed samples are a viable measure to consider in population-level studies, providing estimates of the community-level diversity that are highly correlated with diversity estimates using individually sequenced samples [61–66]. The core microbiomes of both *Podarcis* species intersected in 13 ASVs from several genera commonly found in the gut communities of other lizard species (*Alistipes*, *Bacteroides*, *Breznakia*, *Desulfovibrio*, *Odoribacter*, *Oscillibacter*, *Parabacteroides*, and Ruminococcaceae UBA1819; e.g*.*,[67–69] and also showed a symmetric difference consisting of bacterial taxa with high prevalence levels in either *P. lilfordi* or *P. pityusensis*. In the former we detected 11 species-specific core taxa that could be classified to genus (*Alistipes*, *Anaeroplasma*, *Bacteroides*, *Bacteroides*, *Desulfovibrio*, Rikenellaceae dgA-11 gut group, *Helicobacter*, and *Parabacteroides*). Taxa from these genera were also identified as core members of *P. lilfordi* gut microbiota by [26] excepting *Alistipes*, *Breznakia*, *Oscillibacter*, Ruminococcaceae UBA1819, and Rikenellaceae dgA-11 gut group. *Podarcis pityusensis* also showed a distinctive core taxa assemblage consistent with bacteria genera commonly found in the gut microbiome of other lizard species (*Bacteroides*, *Coprococcus 3*, *Dielma*, *Erysipelatoclostridium*, *Eubacterium*, *Odoribacter*, *Parabacteroides*, *Robinsoniella*, *and Romboutsia*) [42, 67, 70]. All these findings are consistent with the long-term geographic isolation of both *P. lilfordi* and *P. pityusensis* lineages that started *ca.* 5.33 Ma ago and subsequent geographical, ecological and evolutionary divergence [20].

### Factors Shaping the Faecal Microbiota of the Balearic Podarcis Lizards

Extant *P. lilfordi* populations are restricted to coastal islands and islets of Mallorca, Menorca, and the Cabrera archipelago, which acted as refugia after the extinction of mainland populations *ca.* 2000 years ago due to the anthropic introduction of predators and competitors [12, 13]. Such isolation could be even older according to Holocene sea-level data from the western Mediterranean Sea [71]. Therefore, each islet would constitute a particular evolutionary scenario with an independent demographic history and linked to different ecological conditions. Indeed, both morphological and genetic differentiation of *P. lilfordi* populations have led to consider them as independent evolutionary significant units [72]. In this regard, our results suggest that the allopatric status of the Balearic *Podarcis* populations could also have shaped their gut microbiota. Our exploratory PERMANOVA analyses retrieved the islet adscription and main island district as the main factor in explaining bacterial community composition. Consistently, each individual population showed a high proportion of non-shared ASVs (e.g*.*, up to 32.77% of the ASVs found in the Porros population were exclusive from this islet). This result is even more significant if we consider that rarefaction curves of the samples reached the plateau stage, thus ensuring that comparisons are based on a comprehensive view of their respective ASV diversity. All these results reinforce the consideration of every single *Podarcis* population as a unique evolutionary entity hosting a singular gut microbiota.

PERMANOVA analyses based on datasets confirmed by samples from populations with matching collecting seasons: (spring + summer and summer + autumn) allowed us to estimate the effect of the seasons on the gut microbiota of the Balearic *Podarcis*. In general, samples from the same population (island/islet) and collecting season showed similar composition in terms of microbial communities. In this regard, the seasonality of food has been demonstrated to partially affect the diet composition of *P. lilfordi* lizards [73]. Resources availability is usually higher in spring when both arthropod prey and plants are abundant, while summer is characterised by the consumption of vegetal tissues and ants, and autumn is marked by food scarcity [73, 74]. Previous studies have shown diet composition affects the lizard’s gut microbiota [68]. Our results not only point in the same direction but also highlight the importance of the interaction between the specific islets and the seasons of the year, thus suggesting that the different Balearic *Podarcis* populations inhabit unique ecological scenarios.

The evolutionary history of *Podarcis* in the Balearic archipelago is marked by the occurrence of allopatric processes leading to the extant isolated populations that exhibit genetic, morphological, ecological, and ethological differences, in many cases unique to one population [72]. Such isolation is likely to have impeded both the gene flow between populations and the dispersal of gut symbionts, thus favoring the parallel divergence of the hosts and their gut microbiota. This divergence process could have been reinforced by a housing effect similar to that reported from other animal groups where genetically divergent individuals/species inhabiting the same place share more bacteria taxa than individuals living apart [75, 76]. In this regard, the consumption of conspecifics (cannibalism) reported in *P. lilfordi* [17] could facilitate horizontal gut microbiome transmission among sympatric individuals as proposed for other organisms [77]. Environmental bacteria acquired from prey and plant material, or even from coprophagy, are also known to constitute an important fraction of the gut microbiome in lizards [24, 78], which is an islet system that could contribute to homogenizing the bacterial communities of the individuals. In addition, vertical transmission of gut microbiota during birth has been demonstrated in other lizard species [79]. In turn, microbial communities could drive host evolution by influencing key aspects such as adaptation to resource utilisation and behaviour [5, 6, 11], thus consolidating the divergence between lizard lineages/populations. All of the above suggest that allopatric divergence of hosts coupled with both the limited dispersal of gut symbionts and the ecological idiosyncrasy of their isolated habitats could have shaped the faecal microbiota of these two species of endangered lizards of the Balearic Islands.

## Supplementary Information

Below is the link to the electronic supplementary material.

Supplementary file1 (PDF 964 KB)

Supplementary file2 (PDF 3511 KB)

Supplementary file3 (PDF 5977 KB)

Supplementary file4 (PDF 4187 KB)

Supplementary file5 (XLSX 1328 KB)

Supplementary file6 (XLSX 20 KB)

Supplementary file7 (DOCX 35 KB)

Supplementary file8 (XLSX 63 KB)

## Data Availability

Raw sequences are available in the Sequence Read Archive (SRA) database at NCBI under BioProject ID PRJNA693423.
