# Supplementary figures and images for: Faecal Microbiota Divergence in Allopatric Populations of Podarcis lilfordi and P. pityusensis, Two Lizard Species Endemic to the Balearic Islands
Source: Microb Ecol. 2022 Apr 28;85(4):1564–77. doi: 10.1007/s00248-022-02019-3 (PMC10167182; doi:10.1007/s00248-022-02019-3)

**A)**

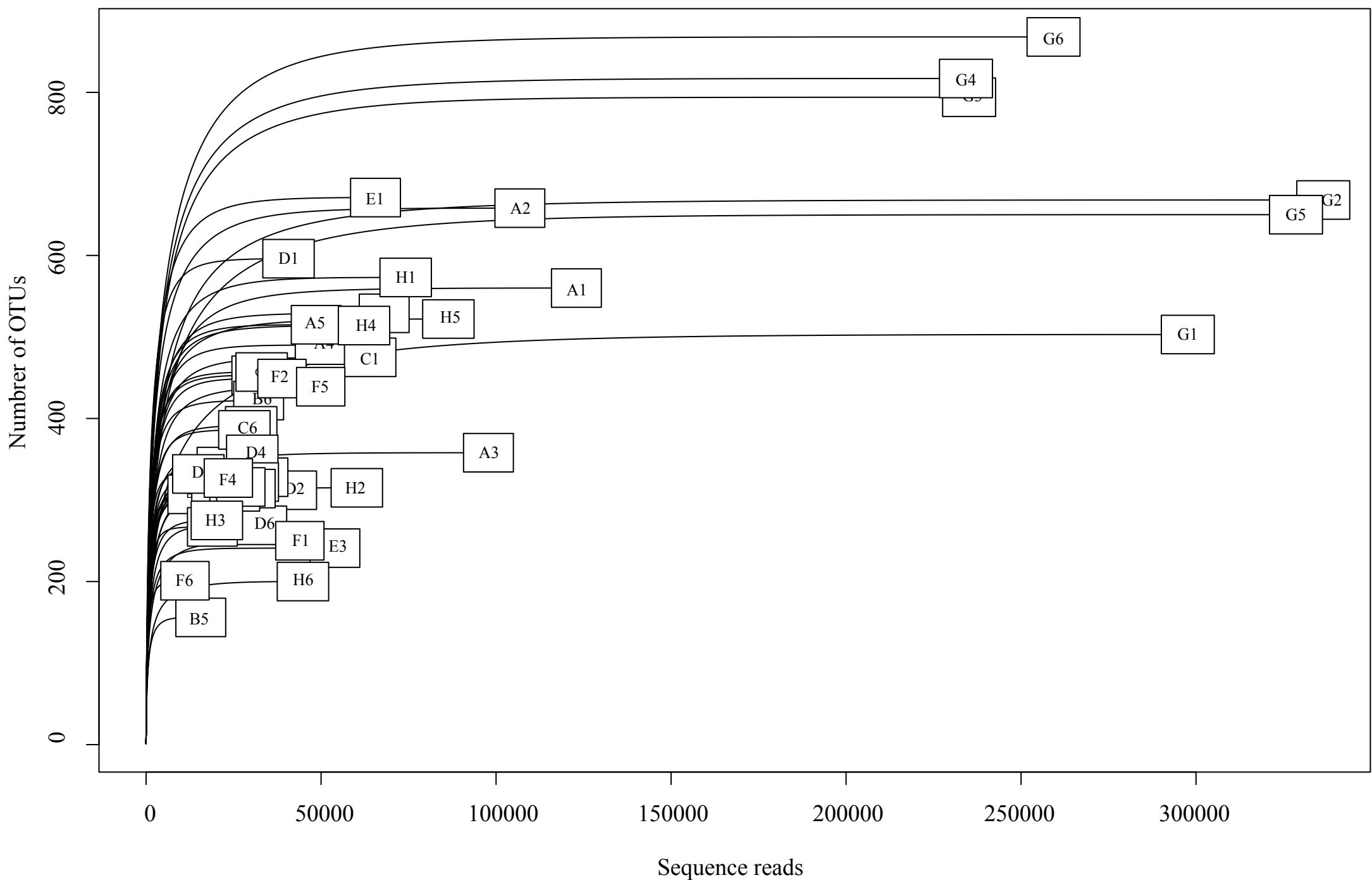

**B)**

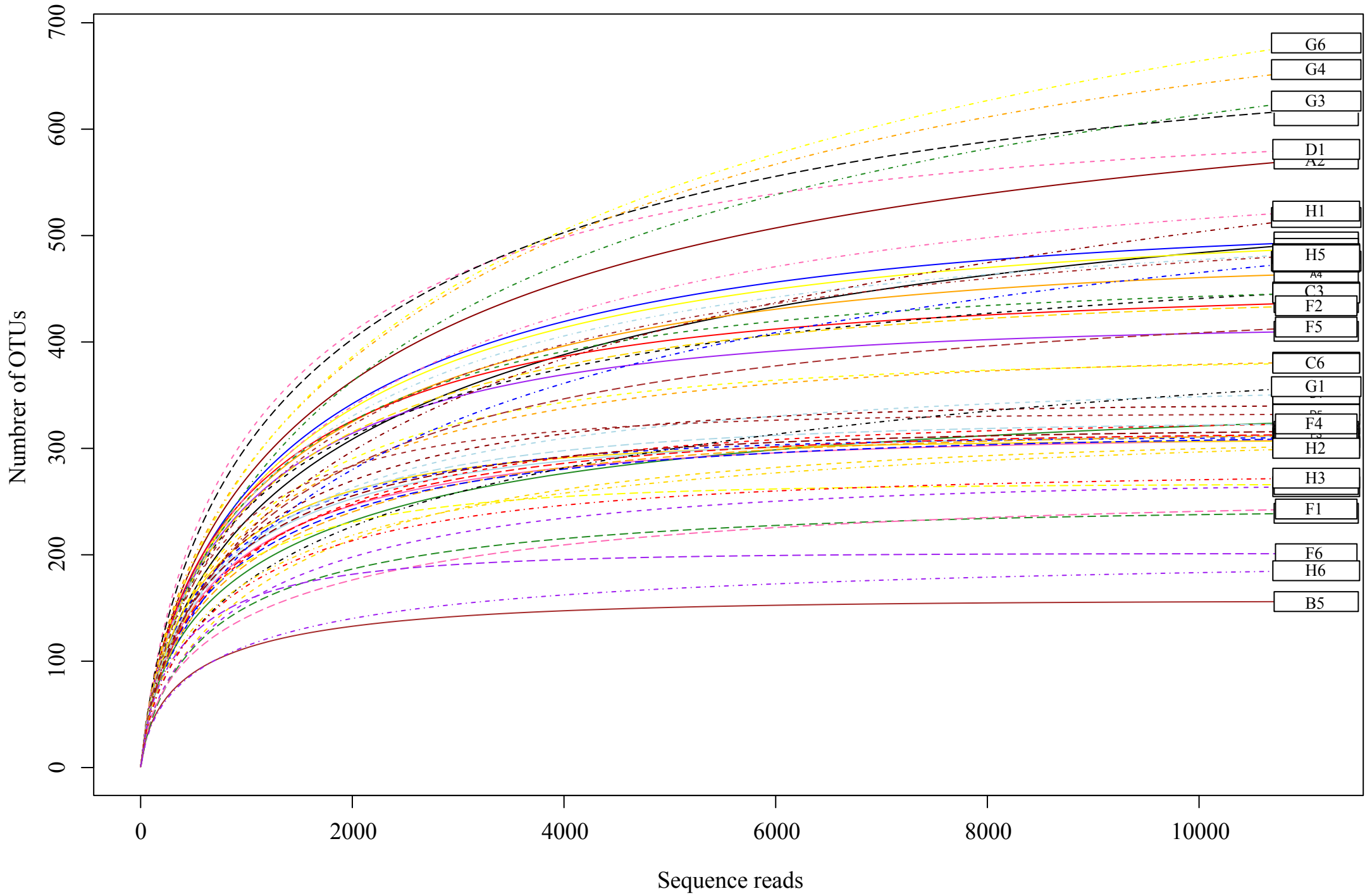

Supplement: Supplementary file 1 — Supplementary file1 (PDF 964 KB) [file 248_2022_2019_MOESM1_ESM.pdf]

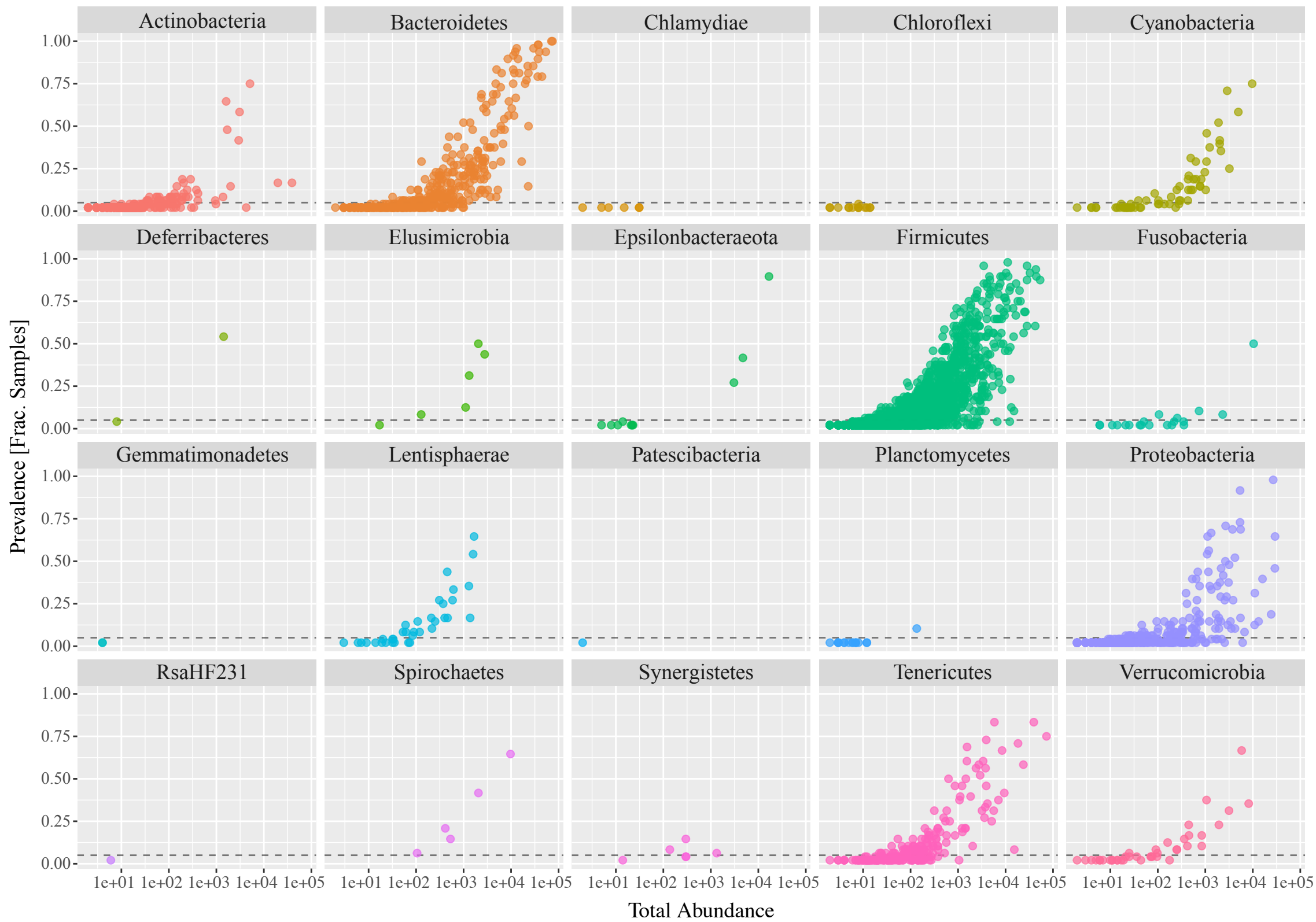

Supplement: Supplementary file 2 — Supplementary file2 (PDF 3511 KB) [file 248_2022_2019_MOESM2_ESM.pdf]

ID

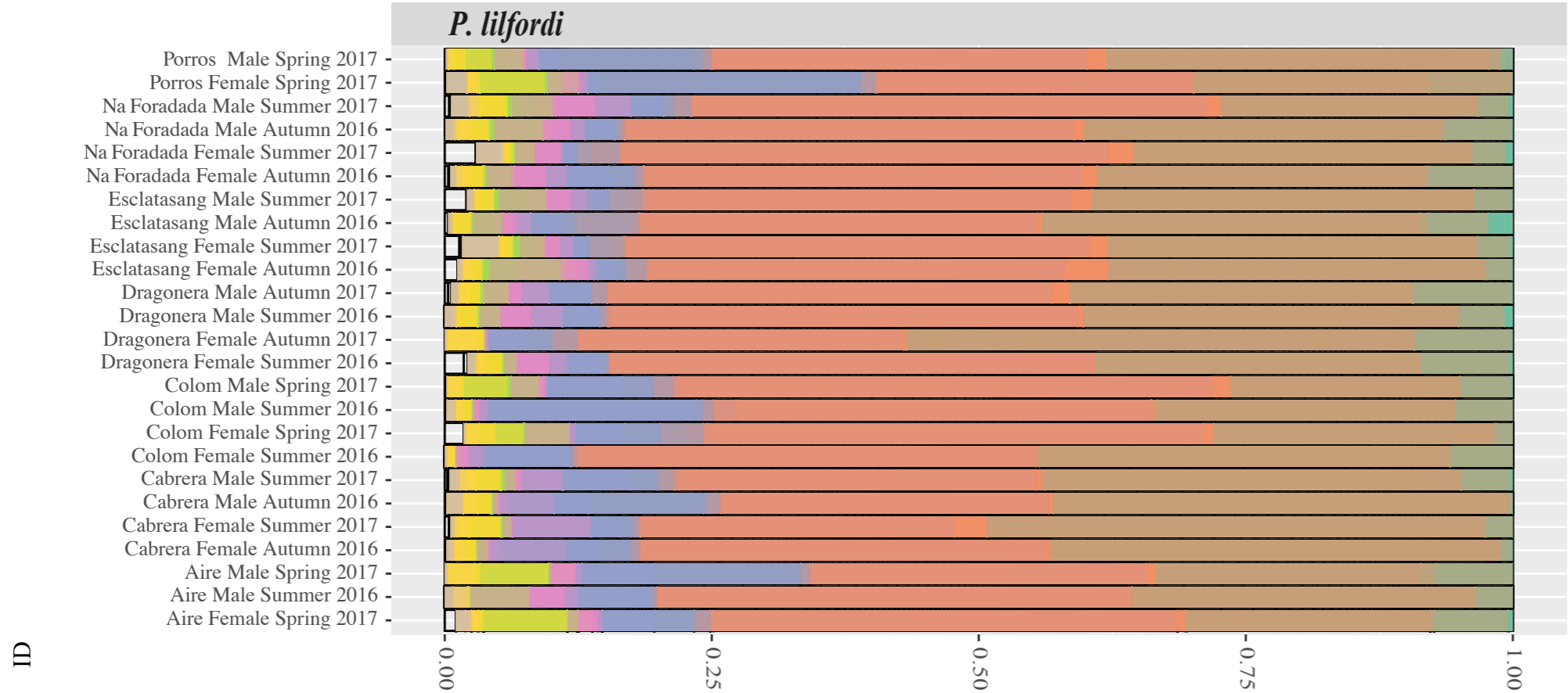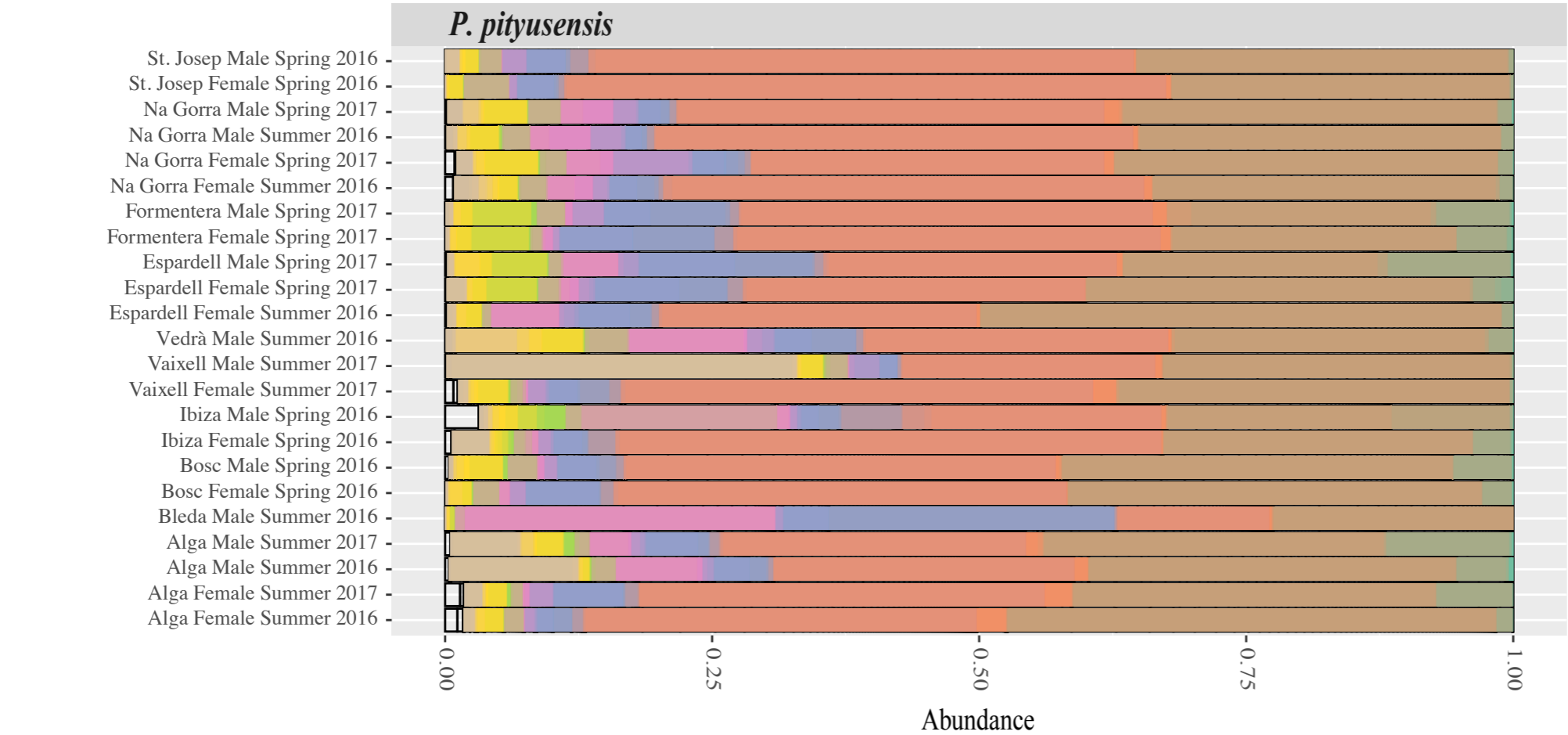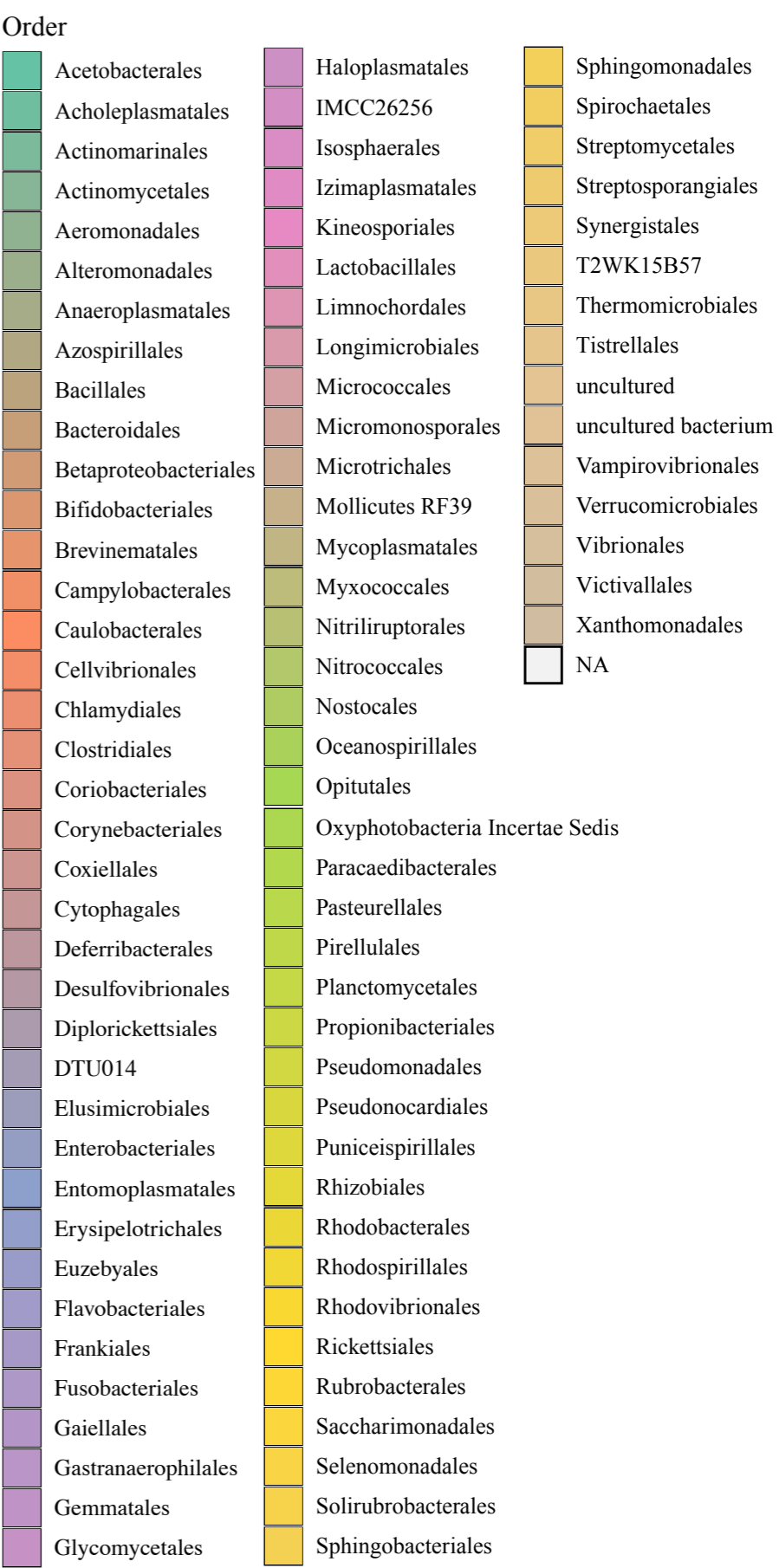

Supplement: Supplementary file 3 — Supplementary file3 (PDF 5977 KB) [file 248_2022_2019_MOESM3_ESM.pdf]

*P. lilfordi*

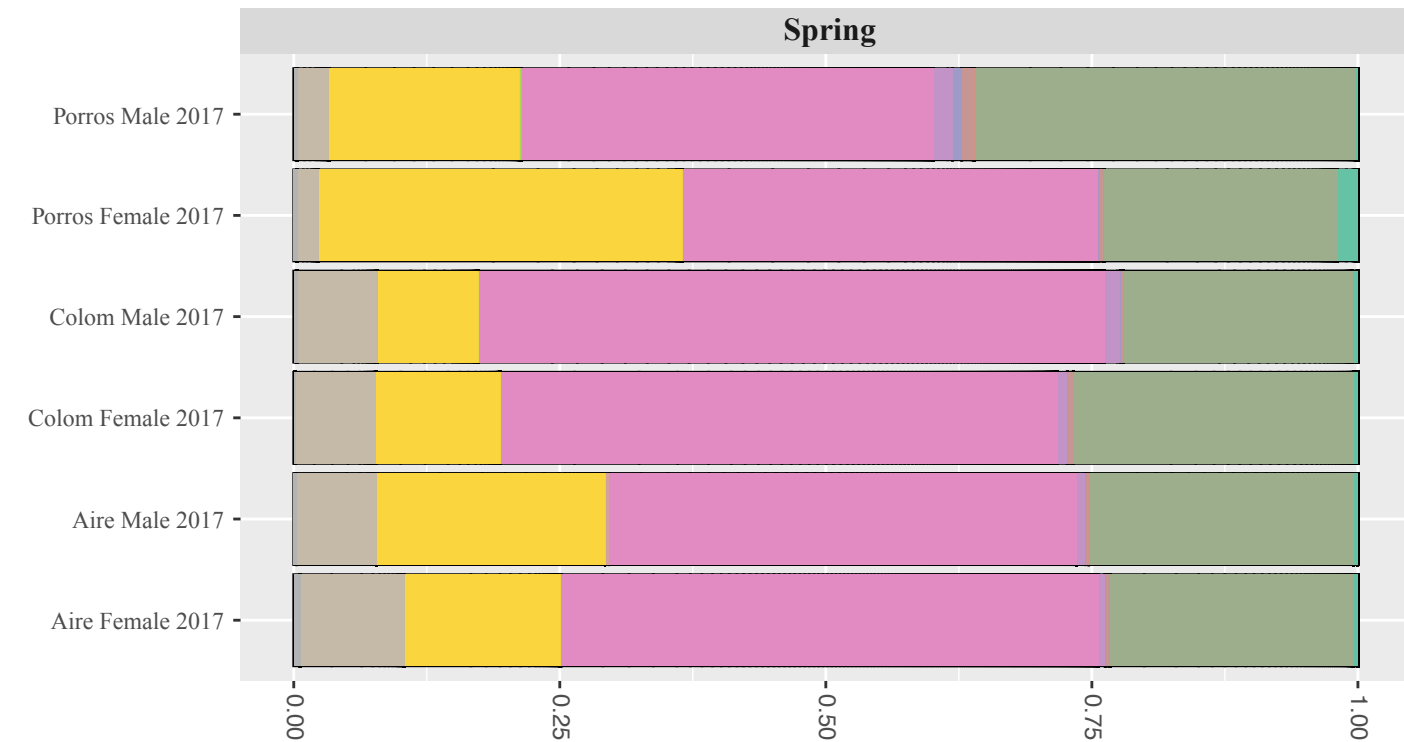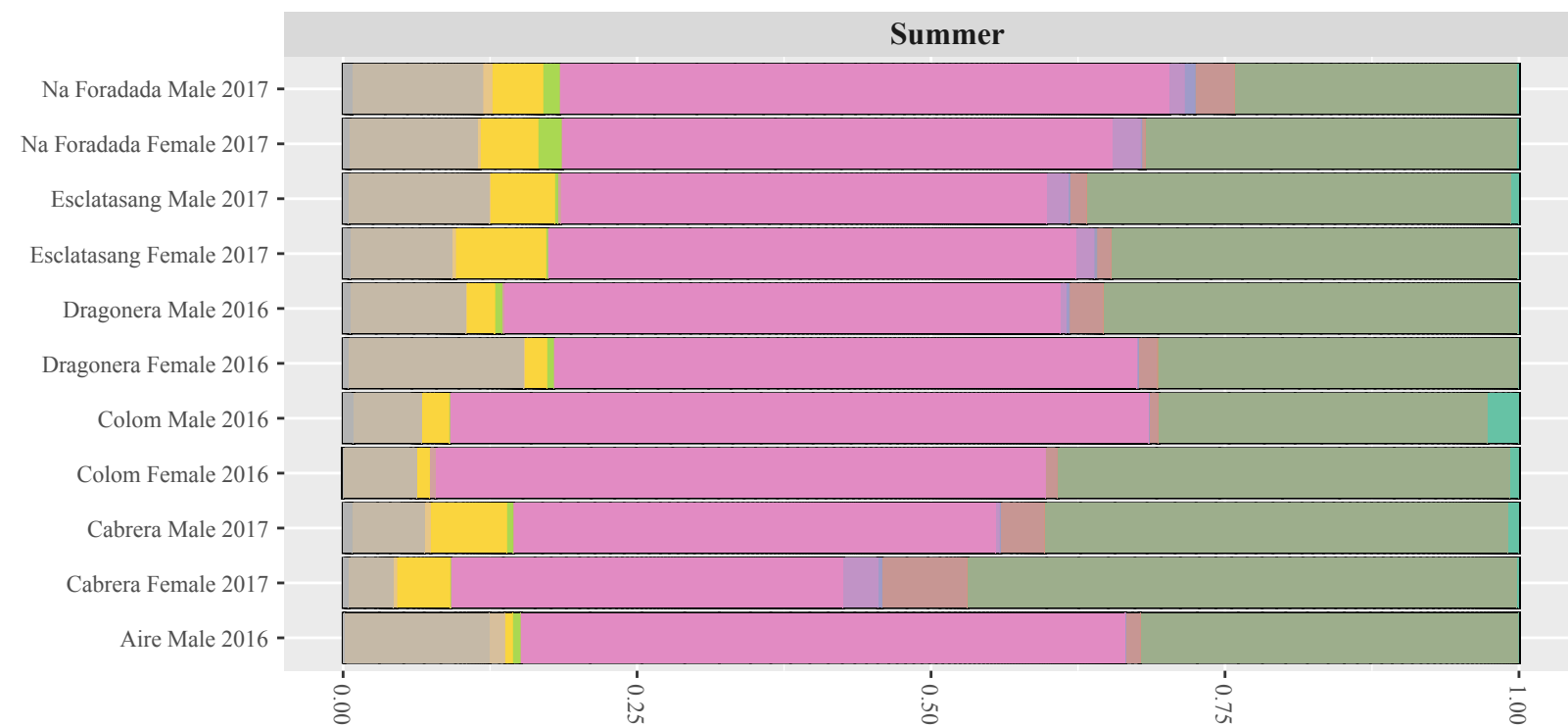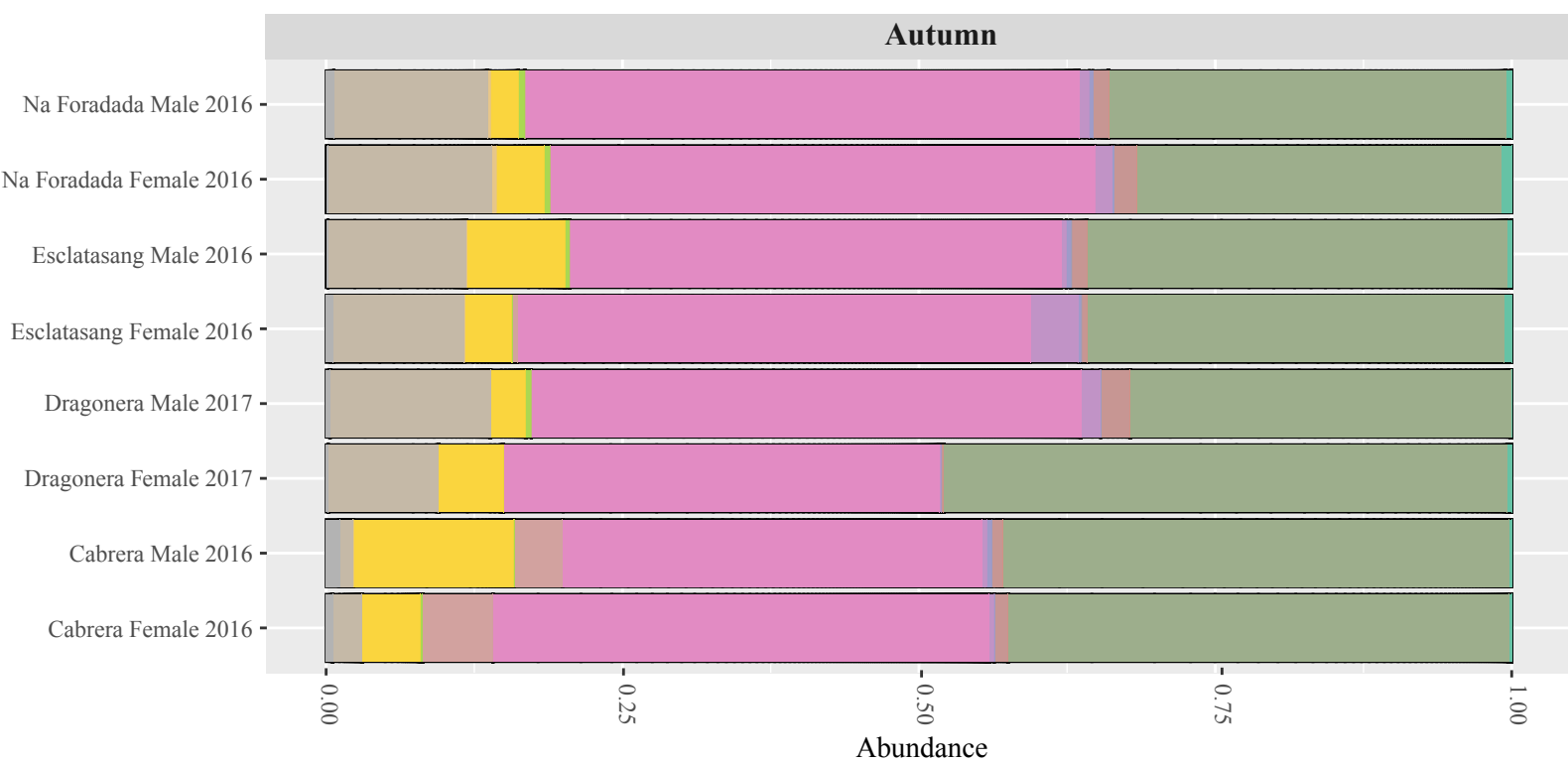

*P. pityusensis*

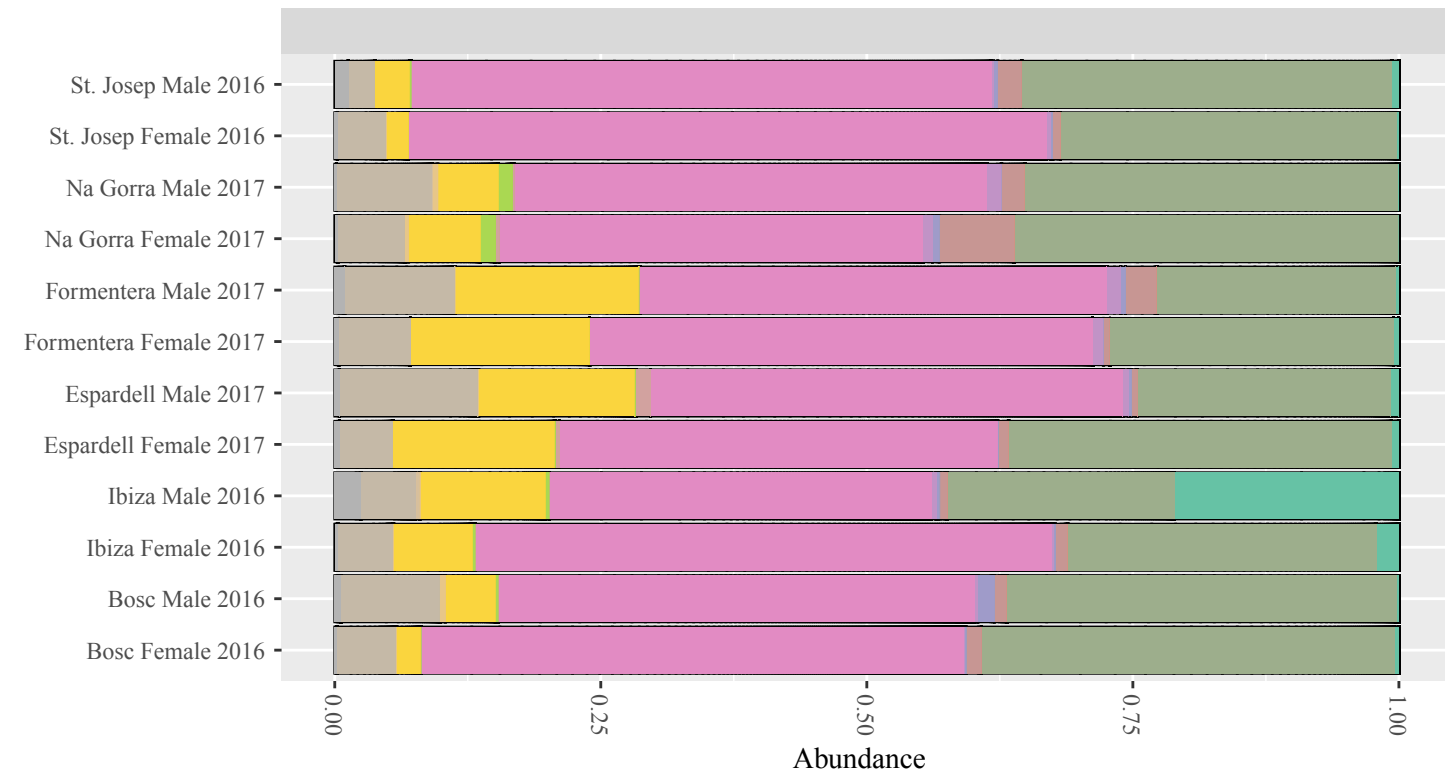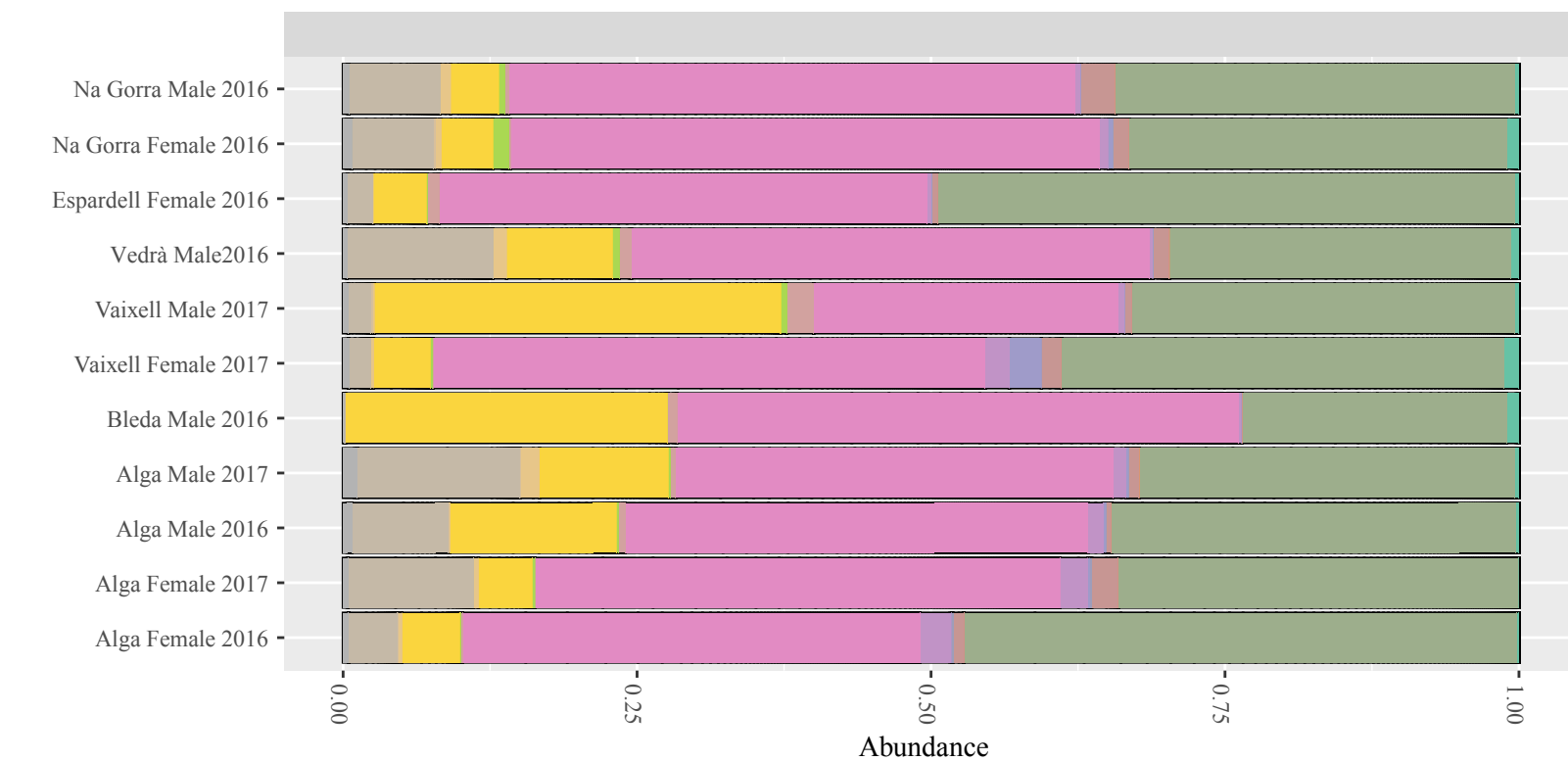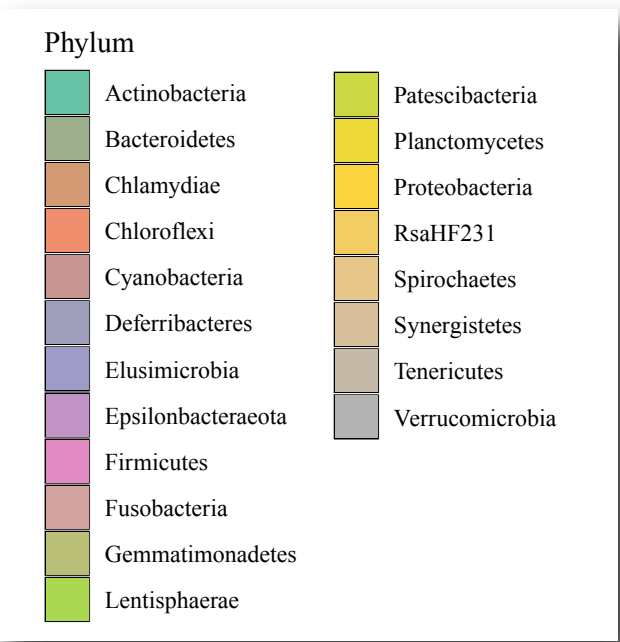

Supplement: Supplementary file 4 — Supplementary file4 (PDF 4187 KB) [file 248_2022_2019_MOESM4_ESM.pdf]
