# Supplementary material for: Faecal Microbiota Divergence in Allopatric Populations of Podarcis lilfordi and P. pityusensis, Two Lizard Species Endemic to the Balearic Islands
Source: Microb Ecol. 2022 Apr 28;85(4):1564–77. doi: 10.1007/s00248-022-02019-3 (PMC10167182; doi:10.1007/s00248-022-02019-3)
Supplement: Supplementary file 7 — Supplementary file7 (DOCX 35 KB) [file 248_2022_2019_MOESM7_ESM.docx]

**Table S3:** Kruskal-Wallis test results based on comparisons of *P. lilfordi vs*. *P. pityusensis* α-diversity indexes.

| **Datasets** | **Diversity analyses** | **signif.level** | **dif.com.obs.dif** | **dif.com.critical.dif** | **dif.com.difference** |
| --- | --- | --- | --- | --- | --- |
| ***P. lilfordi* (all samples) *vs.* *P. pityusensis* (all samples)** | **Observed** | 0.05 | 7.14 | 7.93 | FALSE |
|  | **Chao1** | 0.05 | 7.14 | 7.93 | FALSE |
|  | **Shannon** | 0.05 | 3.88 | 7.93 | FALSE |
|  | **Simpson** | 0.05 | 0.79 | 7.93 | FALSE |
|  | **PD** | 0.05 | 6.55 | 7.93 | FALSE |
| ***P. lilfordi* (spring) *vs.* *P. pityusensis* (spring)** | **Observed** | 0.05 | 6.00 | 5.23 | **TRUE** |
|  | **Chao1** | 0.05 | 6.00 | 5.23 | **TRUE** |
|  | **Shannon** | 0.05 | 4.75 | 5.23 | FALSE |
|  | **Simpson** | 0.05 | 2.50 | 5.23 | FALSE |
|  | **PD** | 0.05 | 6.25 | 5.23 | **TRUE** |
| ***P. lilfordi* (summer) *vs.* *P. pityusensis* (summer)** | **Observed** | 0.05 | 1.64 | 5.43 | FALSE |
|  | **Chao1** | 0.05 | 2.27 | 5.43 | FALSE |
|  | **Shannon** | 0.05 | 0.64 | 5.43 | FALSE |
|  | **Simpson** | 0.05 | 1.55 | 5.43 | FALSE |
|  | **PD** | 0.05 | 1.55 | 5.43 | FALSE |

**Table S4:** Kruskal-Wallis test results based on intra-specific comparisons of α-diversity indexes by collecting season.

| **Species** | **Dataset** | **Alpha diversity** | **chi-squared** | **df** | **p-value** |
| --- | --- | --- | --- | --- | --- |
| ***P. lilfordi*** | **Spring** | **Shannon** | 2.000 | 2 | 0.368 |
|  |  | **Chao1** | 0.000 | 2 | 1.000 |
|  |  | **Observed** | 0.286 | 2 | 0.867 |
|  |  | **Simpson** | 2.000 | 2 | 0.368 |
|  |  | **PD** | 3.714 | 2 | 0.156 |
|  | **Summer** | **Shannon** | 5.546 | 5 | 0.353 |
|  |  | **Chao1** | 5.864 | 5 | 0.320 |
|  |  | **Observed** | 6.046 | 5 | 0.302 |
|  |  | **Simpson** | 5.273 | 5 | 0.384 |
|  |  | **PD** | 5.864 | 5 | 0.320 |
|  | **Autumn** | **Shannon** | 4.667 | 3 | 0.198 |
|  |  | **Chao1** | 5.500 | 3 | 0.139 |
|  |  | **Observed** | 5.102 | 3 | 0.164 |
|  |  | **Simpson** | 2.667 | 3 | 0.446 |
|  |  | **PD** | 6.167 | 3 | 0.104 |
| ***P. pityusensis*** | **Spring** | **Shannon** | 9.308 | 5 | 0.0974 |
|  |  | **Chao1** | 8.077 | 5 | 0.152 |
|  |  | **Observed** | 7.661 | 5 | 0.176 |
|  |  | **Simpson** | 6.462 | 5 | 0.264 |
|  |  | **PD** | 9.000 | 5 | 0.109 |
|  | **Summer** | **Shannon** | 9.455 | 5 | 0.092 |
|  |  | **Chao1** | 6.818 | 5 | 0.235 |
|  |  | **Observed** | 6.818 | 5 | 0.235 |
|  |  | **Simpson** | 9.318 | 5 | 0.097 |
|  |  | **PD** | 6.818 | 5 | 0.235 |

**Table S6:** Results of Mantel tests for correlation between differences in terms of number of lizard pellets pooled per sample and microbiome community composition distances (Weighted and Unweighted UniFrac) for the entire dataset and for each of the subsets of samples merged by season (spring, summer and autumn datasets).

| **Dataset** | **Unweighted UniFrac** | | **Weighted UniFrac** | |
| --- | --- | --- | --- | --- |
|  | **Mantel statistic r** | **Significance** | **Mantel statistic r** | **Significance** |
| **Complete** | -0.0656 | 0.7763 | 0.0931 | 0.1257 |
| **Spring** | -0.0203 | 0.5283 | -0.0045 | 0.5026 |
| **Summer** | -0.1076 | 0.7854 | 0.1212 | 0.1519 |
| **Autumn** | 0.2611 | 0.0969 | -0.1411 | 0.7448 |

**Table S7a:** List of *P. lilfordi* core taxa (90%) at the finest available level of taxonomic resolution of kingdom Bacteria (Shared core with *P. pityusensis* shown in bold).

| ***P. lilfordi*** | | | | |  |
| --- | --- | --- | --- | --- | --- |
| **Phylum** | **Class** | **Order** | **Family** | **Genus** | **Species** |
| **Bacteroidetes** | **Bacteroidia** | **Bacteroidales** | **Bacteroidaceae** | ***Bacteroides*** |  |
| **Bacteroidetes** | **Bacteroidia** | **Bacteroidales** | **Bacteroidaceae** | ***Bacteroides*** |  |
| **Bacteroidetes** | **Bacteroidia** | **Bacteroidales** | **Bacteroidaceae** | ***Bacteroides*** |  |
| **Bacteroidetes** | **Bacteroidia** | **Bacteroidales** | **Bacteroidaceae** | ***Bacteroides*** |  |
| Bacteroidetes | Bacteroidia | Bacteroidales | Bacteroidaceae | *Bacteroides* |  |
| Bacteroidetes | Bacteroidia | Bacteroidales | Bacteroidaceae | *Bacteroides* |  |
| **Bacteroidetes** | **Bacteroidia** | **Bacteroidales** | **Marinifilaceae** | ***Odoribacter*** |  |
| **Bacteroidetes** | **Bacteroidia** | **Bacteroidales** | **Marinifilaceae** | ***Odoribacter*** |  |
| **Bacteroidetes** | **Bacteroidia** | **Bacteroidales** | **Rikenellaceae** | ***Alistipes*** |  |
| Bacteroidetes | Bacteroidia | Bacteroidales | Rikenellaceae | *Alistipes* |  |
| Bacteroidetes | Bacteroidia | Bacteroidales | Rikenellaceae | *dgA-11 gut group* |  |
| **Bacteroidetes** | **Bacteroidia** | **Bacteroidales** | **Tannerellaceae** | ***Parabacteroides*** |  |
| Bacteroidetes | Bacteroidia | Bacteroidales | Tannerellaceae | *Parabacteroides* |  |
| Epsilonbacteraeota | Campylobacteria | Campylobacterales | Helicobacteraceae | *Helicobacter* |  |
| Firmicutes | Clostridia | Clostridiales | Lachnospiraceae |  |  |
| **Firmicutes** | **Clostridia** | **Clostridiales** | **Ruminococcaceae** | ***Oscillibacter*** |  |
| **Firmicutes** | **Clostridia** | **Clostridiales** | **Ruminococcaceae** | ***UBA1819*** |  |
| **Firmicutes** | **Clostridia** | **Clostridiales** |  |  |  |
| Firmicutes | Clostridia | Clostridiales |  |  |  |
| Firmicutes | Clostridia | Clostridiales |  |  |  |
| **Firmicutes** | **Erysipelotrichia** | **Erysipelotrichales** | **Erysipelotrichaceae** | ***Breznakia*** |  |
| **Proteobacteria** | **Deltaproteobacteria** | **Desulfovibrionales** | **Desulfovibrionaceae** | ***Desulfovibrio*** | ***desulfuricans*** |
| Proteobacteria | Deltaproteobacteria | Desulfovibrionales | Desulfovibrionaceae | *Desulfovibrio* |  |
| Tenericutes | Mollicutes | Anaeroplasmatales | Anaeroplasmataceae | *Anaeroplasma* |  |

**Table S7b:** List of *P. pityusensis* core taxa (90%) at the finest available level of taxonomic resolution of kingdom Bacteria (Shared core with *P. lilfordi* shown in bold).

| ***P. pityusensis*** | | | | |  |
| --- | --- | --- | --- | --- | --- |
| **Phylum** | **Class** | **Order** | **Family** | **Genus** | **Species** |
| **Bacteroidetes** | **Bacteroidia** | **Bacteroidales** | **Bacteroidaceae** | ***Bacteroides*** |  |
| **Bacteroidetes** | **Bacteroidia** | **Bacteroidales** | **Bacteroidaceae** | ***Bacteroides*** |  |
| **Bacteroidetes** | **Bacteroidia** | **Bacteroidales** | **Bacteroidaceae** | ***Bacteroides*** |  |
| **Bacteroidetes** | **Bacteroidia** | **Bacteroidales** | **Bacteroidaceae** | ***Bacteroides*** |  |
| Bacteroidetes | Bacteroidia | Bacteroidales | Bacteroidaceae | *Bacteroides* |  |
| Bacteroidetes | Bacteroidia | Bacteroidales | Bacteroidaceae | *Bacteroides* |  |
| Bacteroidetes | Bacteroidia | Bacteroidales | Bacteroidaceae | *Bacteroides* |  |
| **Bacteroidetes** | **Bacteroidia** | **Bacteroidales** | **Marinifilaceae** | ***Odoribacter*** |  |
| **Bacteroidetes** | **Bacteroidia** | **Bacteroidales** | **Marinifilaceae** | ***Odoribacter*** |  |
| Bacteroidetes | Bacteroidia | Bacteroidales | Marinifilaceae | *Odoribacter* |  |
| **Bacteroidetes** | **Bacteroidia** | **Bacteroidales** | **Rikenellaceae** | ***Alistipes*** |  |
| **Bacteroidetes** | **Bacteroidia** | **Bacteroidales** | **Tannerellaceae** | ***Parabacteroides*** |  |
| Bacteroidetes | Bacteroidia | Bacteroidales | Tannerellaceae | *Parabacteroides* |  |
| Firmicutes | Clostridia | Clostridiales | Eubacteriaceae | *Eubacterium* |  |
| Firmicutes | Clostridia | Clostridiales | Lachnospiraceae | *Coprococcus 3* |  |
| Firmicutes | Clostridia | Clostridiales | Lachnospiraceae | *Robinsoniella* |  |
| Firmicutes | Clostridia | Clostridiales | Lachnospiraceae |  |  |
| Firmicutes | Clostridia | Clostridiales | Peptostreptococcaceae | *Romboutsia* |  |
| **Firmicutes** | **Clostridia** | **Clostridiales** | **Ruminococcaceae** | ***Oscillibacter*** |  |
| **Firmicutes** | **Clostridia** | **Clostridiales** | **Ruminococcaceae** | ***UBA1819*** |  |
| Firmicutes | Clostridia | Clostridiales | Ruminococcaceae |  |  |
| **Firmicutes** | **Clostridia** | **Clostridiales** |  |  |  |
| Firmicutes | Clostridia | Clostridiales |  |  |  |
| **Firmicutes** | **Erysipelotrichia** | **Erysipelotrichales** | **Erysipelotrichaceae** | ***Breznakia*** |  |
| Firmicutes | Erysipelotrichia | Erysipelotrichales | Erysipelotrichaceae | *Dielma* |  |
| Firmicutes | Erysipelotrichia | Erysipelotrichales | Erysipelotrichaceae | *Erysipelatoclostridium* |  |
| **Proteobacteria** | **Deltaproteobacteria** | **Desulfovibrionales** | **Desulfovibrionaceae** | ***Desulfovibrio*** | ***desulfuricans*** |
